# Assembly dynamics and structure of an aegerolysin, ostreolysin A6

**DOI:** 10.1016/j.jbc.2023.104940

**Published:** 2023-06-19

**Authors:** Neval Yilmaz, Anastasija Panevska, Nario Tomishige, Ludovic Richert, Yves Mély, Kristina Sepčić, Peter Greimel, Toshihide Kobayashi

**Affiliations:** 1 Lipid Biology Laboratory, RIKEN, 2-1, Wako, Saitama, Japan; 2 NanoLSI, Kanazawa University, Kakuma-machi, Kanazawa, Ishikawa, Japan; 3 Department of Biology, Biotechnical Faculty, University of Ljubljana, Ljubljana, Slovenia; 4 Laboratoire de Bioimagerie et Pathologies, UMR 7021 CNRS, Université de Strasbourg, Faculté de Pharmacie, Illkirch, France

**Keywords:** high-speed atomic force microscopy, aegerolysin, ostreolysin, ceramide phosphoethanolamine, supported lipid bilayer

## Abstract

Ostreolysin A6 (OlyA6) is an oyster mushroom-derived membrane-binding protein that, upon recruitment of its partner protein, pleurotolysin B, forms a cytolytic membrane pore complex. OlyA6 itself is not cytolytic but has been reported to exhibit pro-apoptotic activities in cell culture. Here we report the formation dynamics and the structure of OlyA6 assembly on a lipid membrane containing an OlyA6 high-affinity receptor, ceramide phosphoethanolamine, and cholesterol. High-speed atomic force microscopy revealed the reorganization of OlyA6 dimers from initial random surface coverage to 2D protein crystals composed of hexameric OlyA6 repeat units. Crystal growth took place predominantly in the longitudinal direction by the association of OlyA6 dimers, forming a hexameric unit cell. Molecular-level examination of the OlyA6 crystal elucidated the arrangement of dimers within the unit cell and the structure of the dimer that recruits pleurotolysin B for pore formation.

Aegerolysins (Pfam 06,355; InterPro IPR009413) are ∼13 to 20 kDa single domain beta-sandwich folded proteins, which are distributed widely in bacteria and fungi (1). Although more than 400 proteins are reported in this family, knowledge of their activity and biological role is limited. Asp-hemolysin, the first reported aegerolysin protein, induces hemolysis (2). *Pleurotus*-derived aegerolysins themselves are not cytotoxic. However, upon co-oligomerization with a larger ∼59 kDa *Pleurotus* sp. protein (pleurotolysin B; PlyB), bearing a membrane-attack-complex/perforin (MACPF) domain, a stable 13-metric bi-component transmembrane pore is generated in targeted membranes (3). Antitumoral, antiproliferative, and antibacterial activities of aegerolysins are also reported (4). Upregulation of certain aegerolysins has been reported during the development of the producing organisms, suggesting the involvement of aegerolysins in growth and/or development (4, 5, 6, 7). A number of aegerolysins exhibit affinity toward specific lipids and/or lipid membranes (4, 8, 9, 10, 11, 12), raising the potential to employ aegerolysins as useful tools to visualize the localization of cellular lipids (13, 14, 15, 16, 17).

Ostreolysin A6 (PDB: 6MYI; OlyA6) (18) is an oyster mushroom (*Pleurotus* sp.)-derived aegerolysin. OlyA6 and related proteins of ostreolysin A (NCBI: AAX21097.1; OlyA) (4) and rOly (NCBI: KDQ25828.1) (19) (Fig. 1) are reported to enhance fruiting initiation in oyster mushrooms (20), induce plasmalemmal vesicle shedding from cultured mammalian cells (21) and exhibit antiproliferative and pro-apoptotic effects on colon cancer cells (19) as well as ameliorate hepatic steatosis (22). To date, the molecular mechanisms of these activities have not been fully elucidated.

**Figure 1:**
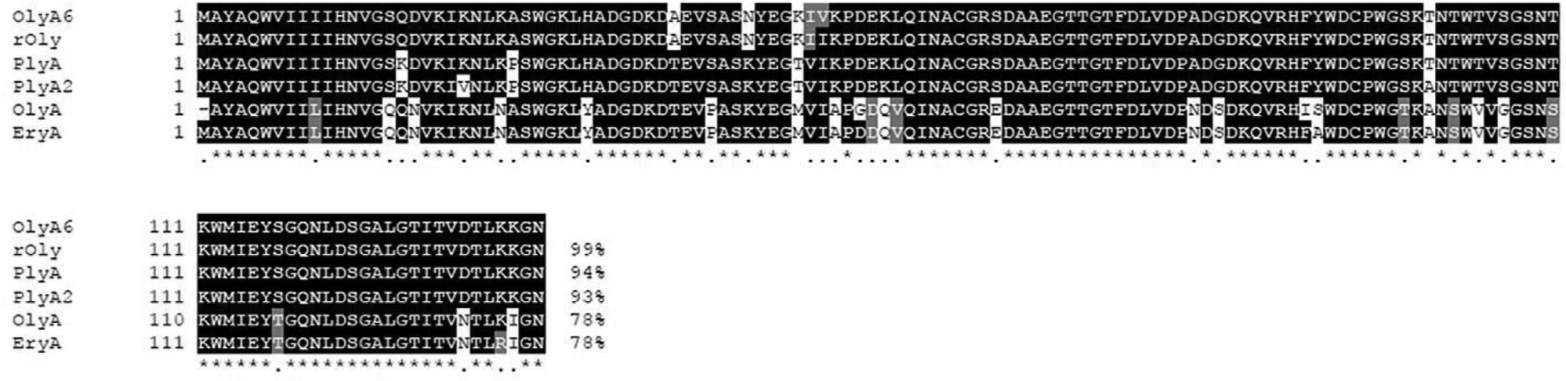
**Boxshade presentations of amino-acid sequence alignments of ostreolysin A6 (OlyA6, UniProtKB/Swiss-Prot: P83467.2), rOly (NCBI KDQ25828.1), pleurotolysin A (PlyA; NCBI BAD66666), ostreolysin A (OlyA, NCBI AAX21097.1) from *Pleurotus ostreatus*, and pleurotolysin A2 (PlyA2, NCBI BAN83906.1) and erylysin A (EryA, NCBI BAI45247.1) from *Pleurotus eryngii*, using the ClustalW (strict).** Positions of the highest similarity are shaded in *black*.

Previous reports described binding of OlyA6, OlyA, and similar proteins from *Pleurotus ostreatus*, pleurotolysin A (PlyA; PDB: 4OEB A) (3, 23), and from *Pleurotus eryngii*, pleurotolysin A2 (PlyA2; NCBI: BAN83906.1) (9), to a mixture of sphingomyelin (SM) and cholesterol (Chol) (3, 9, 15, 18). More recently, 1000-fold higher affinity of these proteins as well as erylysin (EryA; NCBI: BAI45247.1) (24) to lipid membranes composed of ceramide phosphoethanolamine (CPE)/Chol compared to SM/Chol has been reported (10). CPE is an analog of SM, which is found only in trace amounts in mammalian cells but in larger amounts (1–5 mol%) in invertebrates such as *Drosophila* (25) and in some protozoa parasites such as *Trypanosoma brucei* (26) (reviewed in (27)).

In this study, we examined the interaction of OlyA6 with a CPE/Chol membrane by high-speed atomic force microscopy (HS-AFM) and revealed the assembly dynamics and organization of two dimensional (2D) OlyA6 crystals on a CPE/Chol membrane. To gain a more atomistic understanding, we performed molecular dynamics (MD) simulations based on high-resolution AFM topology images. Our results suggest that the ability of the OlyA6 dimers to reorganize from random to an ordered close-packed arrangement may be involved in the biological activity of OlyA6.

## Results and discussion

OlyA6 has been reported to strongly bind to CPE/Chol (1:1) membranes (K_D_ = 1.3 nM) (10). Naturally occurring CPE has a very high gel-to-liquid crystalline phase transition temperature (59 °C for milk CPE (28, 29)) and does not form stable bilayers alone (28). Our initial attempts to prepare supported lipid bilayers (SLBs) using milk CPE/Chol mixtures failed due to inefficient fusion of the CPE/Chol mixture on the mica surface. We hypothesized that synthetic d17:1 (chain length of sphingosine)/12:0 (chain length of fatty acid) CPE with a phase transition temperature of 38 °C (28, 29) would be more suitable to form SLBs. Indeed, d17:1/12:0-CPE/Chol (1:1) liposomes yielded stable planar lipid bilayers on the mica surface and thus were used in all HS-AFM experiments.

### OlyA6 assembles into a 2D crystal on SLB as revealed by HS-AFM

**Figure 2:**
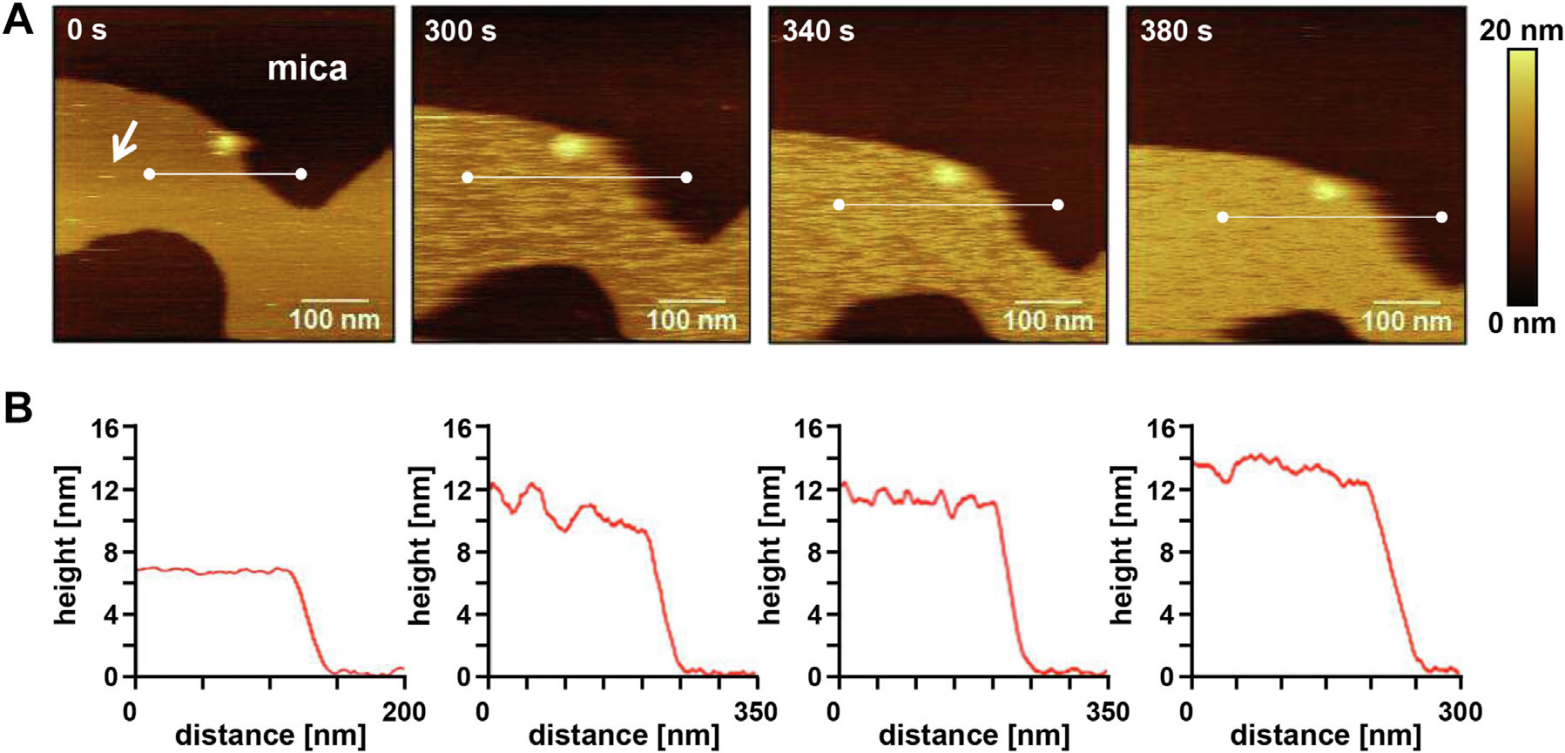
**Binding of OlyA6 to CPE/Chol (1:1) membrane.***A*, HS-AFM height images showing the binding of OlyA6 to the d17:1/12:0-CPE/Chol (1:1) membrane. 0 s was set arbitrarily after the addition of OlyA6 into the imaging medium. *White arrow*, rapidly moving OlyA6. *B*, height profiles along the *white lines* in (*A*) relative to the mica support.

The binding dynamics of OlyA6 was observed *in situ* by HS-AFM at a time interval of 2 s per frame (Fig. 2*A*). After the formation of a stable SLB (yellow-brown) on mica (dark-brown), OlyA6 was added to the imaging medium. Initially, OlyA6 molecules were highly mobile on the membrane (Fig. 2*A*, white arrow) and appeared to diffuse along the membrane surface. OlyA6 was not observed on mica. Within 380 s OlyA6 covered the entire membrane surface. The height profile of membrane-associated OlyA6 was assessed at different levels of surface coverage (Fig. 2*B*). At 0 s the membrane height was 7 nm. The considerable membrane thickness might be caused by the rigid nature of d17:1/12:0-CPE (28) at room temperature. The partial coverage of the membrane surface with OlyA6 at 300 and 340 s resulted in an increase in height to almost 12 nm. At full membrane coverage (380 s), the height of the whole assembly increased further by an additional ∼1 to 1.5 nm, which is potentially caused by a tighter packing and hence a more upright orientation of the OlyA6 molecules at high surface coverage. Taking an OlyA6 monomer height of ∼5 nm into consideration, based on the crystal structure of OlyA6 (15) and homologous PlyA (23), these results suggest that the membrane attachment of OlyA6 is not associated with significant membrane insertion.

**Figure 3:**
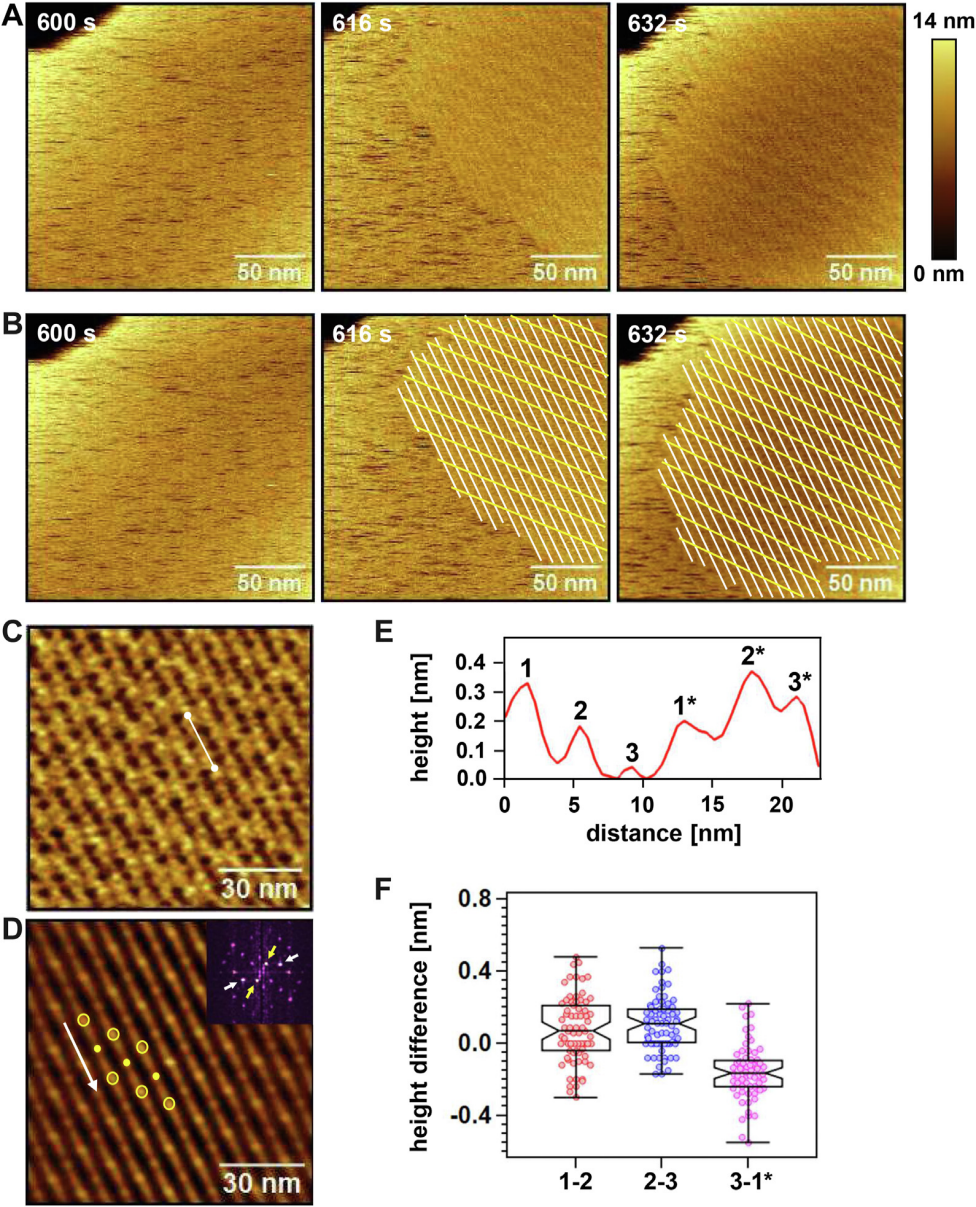
**Assembly of OlyA6 on CPE/Chol (1:1) membrane.***A*, HS-AFM height images showing the rearrangement of OlyA6 from random to crystalline organization on the d17:1/12:0-CPE/Chol (1:1) membrane. These HS-AFM images were recorded following those in Figure 2*A*. *B*, HS-AFM images in Figure 3*A* with *white lines* for OlyA6 crystal stripes and *yellow lines* for the second periodic structure. *C*, Gaussian-filtered HS-AFM height image. *D*, autocorrelated HS-AFM height image with FFT image (inset). *White arrow*, OlyA6 crystal stripe; *yellow open circles*, elevated points constituting the second periodic structure; *yellow filled circles*, lowest height points along the OlyA6 stripes; *white* and *yellow arrows* in FFT image, OlyA6 crystal stripe and second periodic structure, respectively. *E*, height profile along the *white line* in (*C*). Numbers 1, 2, and 3 indicate each subsequent protrusion. The star mark denotes the second repeat unit along the *white line*. *F*, notched box plots showing the distribution for the height difference between the subsequent protrusions with 95% confidence interval of the median. The *center lines* indicate the median values, which are 0.07 (n = 71), 0.11 (n = 71), and −0.17 nm (n = 64) for 1 to 2, 2 to 3, and 3 to 1^∗^, respectively. 1 to 2, 2 to 3, and 3 to 1^∗^ indicate the height of protrusion 1 relative to protrusion 2, height of 2 relative to 3, and height of 3 relative to 1^∗^, respectively. Whiskers display the minimum and maximum values.

Higher resolution imaging of membrane-bound OlyA6 at full surface coverage revealed the gradual rearrangement of OlyA6 from random association to an ordered superstructure (Fig. 3, *A* and *B* and Movie S1). This ordered superstructure consisted of close-packed stripes of OlyA6 crystals (Fig. 3*B*, white lines). In addition to the formation of a periodic structure by OlyA6 stripes (white lines), a second periodic structure appeared simultaneously in the regions of the membrane covered with OlyA6 crystal (Fig. 3*B*, yellow lines). This second structure implies a periodic height change along the stripes. Both structures are easily distinguished in the Gaussian-filtered and the autocorrelated height images (Fig. 3, *C* and *D*). In Figure 3*D*, the OlyA6 stripe direction is indicated by the white arrow, and the features corresponding to the second periodic structure are marked by yellow circles. The Fast Fourier transform (FFT) analysis (Fig. 3*D*, inset) yielded a periodicity of 6.5 nm (white arrows) for the crystal stripes and 14.5 nm (yellow arrows) for the second structure. The height profile along the white line in Figure 3*C* shows the height variation between two points located on the second periodic structure (Fig. 3*E*). Based on our high-resolution AFM images and MD simulation (discussed in detail below) we know that the OlyA6 monomers or dimers appearing as protrusions 1, 2, and 3 in Figure 3*E* constitute one repeat unit. Accordingly, protrusions 1^∗^-3^∗^ constitute the second repeat unit. In these two consecutive repeat units, protrusion 3, not protrusion 3^∗^, exhibits the lowest height. The lowering of protrusion 3 in alternate repeat units generates the second periodicity in height images. The analysis of the height difference between sequential protrusions along the OlyA6 stripes (Fig. 3*F*) yielded median values larger than 0.1 nm for protrusions 2 to 3 and 1^∗^ to 3, suggesting that protrusion 3 is lower than protrusions 2 and 1^∗^. However, this height difference would be smaller or larger in alternate repeat units due to the periodic height fluctuations along the OlyA6 stripes.

**Figure 4:**
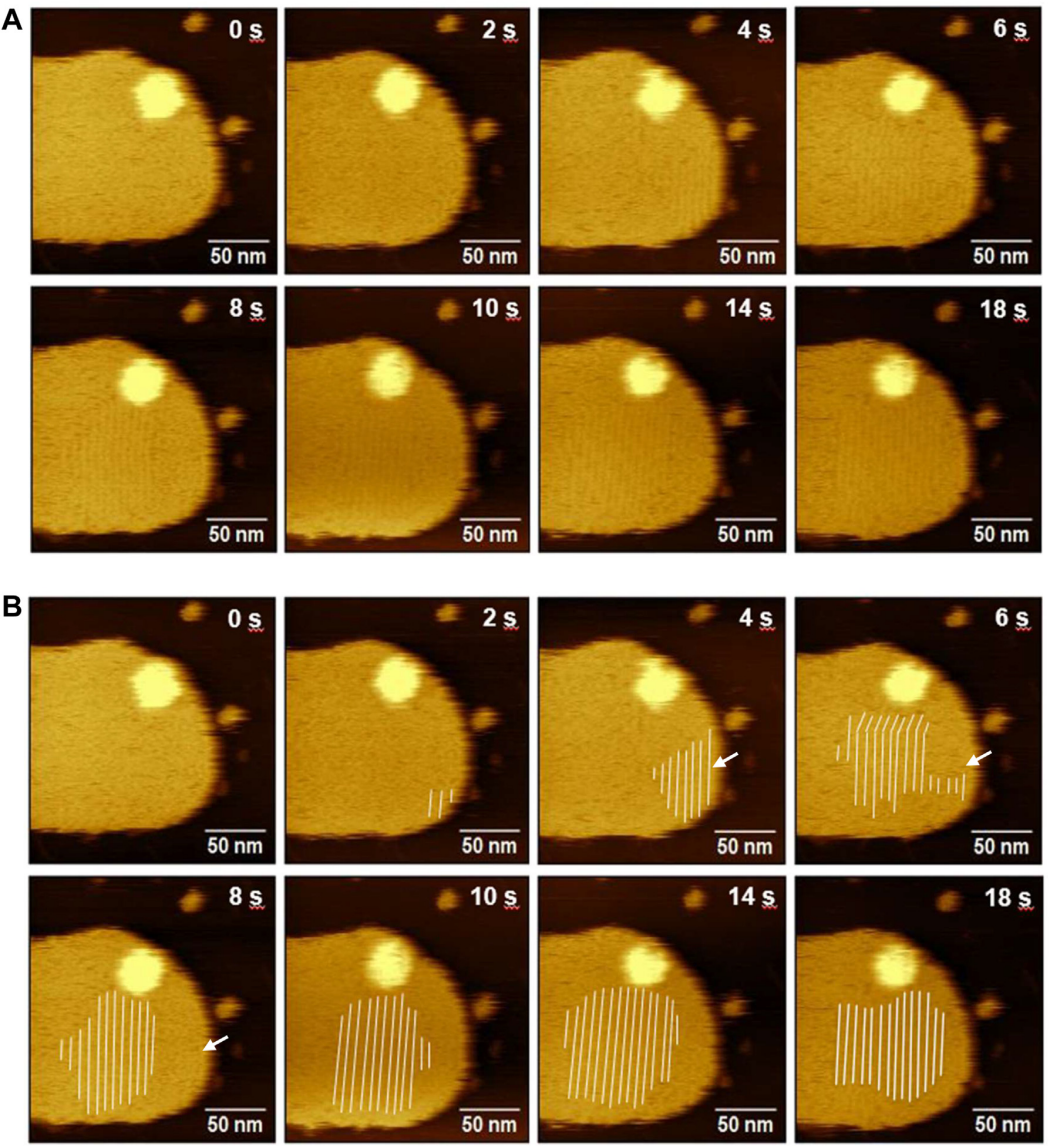
**Assembly dynamics of OlyA6 on CPE/Chol (1:1) membrane.***A*, HS-AFM height images showing the dynamics of OlyA6 assembly on d17:1/12:0-CPE/Chol (1:1) membrane. 0 s was set arbitrarily after addition of OlyA6 into the imaging medium. *B*, HS-AFM images in Figure 4*A* with *white lines* for OlyA6 crystal stripes.

The OlyA6 crystals which grew on the more central region of the membrane were more stable than those at the membrane edge (Fig. 4*A*). This is in contrast to the SM-binding proteins, lysenin (Lys) and equinatoxin II (EqtII) (30, 31, 32), which initiate assembly at the domain edge. Lys and EqtII recognize neither the exposed distal methyl group of the SM phosphocholine head (33) nor the lipid glycerol backbone (34, 35). Both preferentially bind to SM at the domain edge as the lipid head group is more exposed at this region of the membrane. On the contrary, OlyA6 does not require loose lipid packing or exposed lipid head groups. Recent reports showed that OlyA6 interacts with ceramide aminophosphonate (CAEP)-containing membranes as strongly as it does with CPE-containing membranes (12). In CAEP, ethanolamine is conjugated to the phosphorus atom *via* a direct C-P bond, instead of the phosphoester (C-O-P) bond found in CPE. This indicates that the shortened distance between the phosphor atom and the amino group in CAEP does not negatively affect OlyA6 binding, instead may potentially be facilitating its binding.

**Figure 5:**
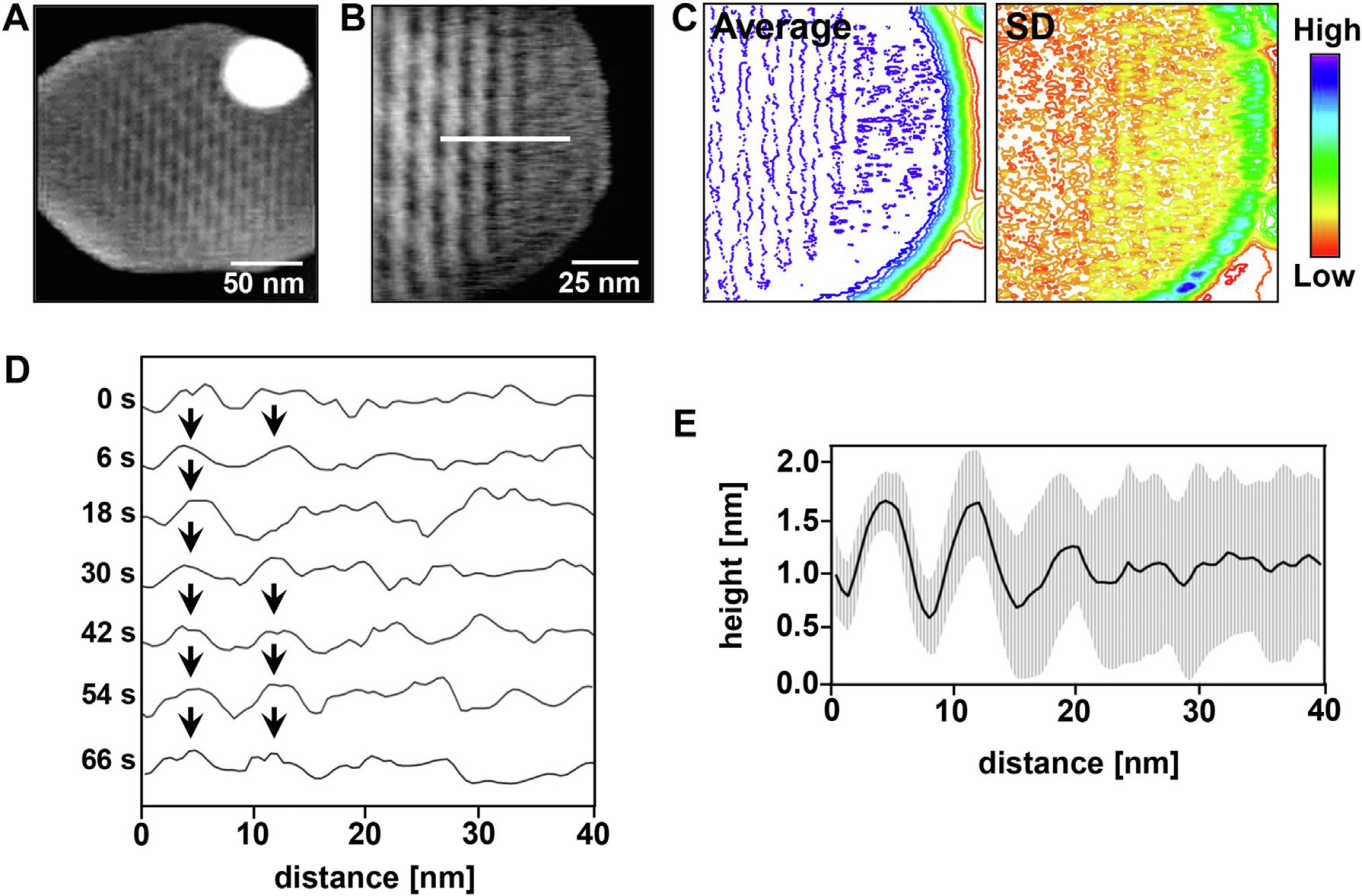
**Stability of OlyA6 assembly on CPE/Chol (1:1) membrane.***A* and *B*, averaged HS-AFM height images showing the stability of OlyA6 assembly on the central region and edge of d17:1/12:0-CPE/Chol (1:1) membrane. *C*, contour maps of average and standard deviation (SD) of pixel height values, corresponding to the data set in (*B*), for 21 sequential frames recorded within a time range of 74 s. *D*, temporal variation in height profile along the *white line* in (*B*). *E*, average height (*solid line*) and standard deviation in height (*shaded region*) along the *white line* in (*B*).

Close to the membrane edge, transient OlyA6 stripes were detected, which dissociated easily, as indicated by arrows in Figure 4*B*. However, it cannot be ruled out that OlyA6 stripes forming at the edge of the membrane diffuse as an oligomeric assembly to the interior of the membrane, facilitating crystal growth on the central region of the membrane. The formation of a stable assembly in the central region of the membrane was also confirmed by the averaged HS-AFM images (Fig. 5, *A* and *B*). In agreement, the standard deviation (SD) contour map showed larger fluctuations in pixel height values for the regions close to the membrane edge (Fig. 5*C*). Height profiles were extracted along the white line in Figure 5*B* to follow the time-dependent change in assembly dynamics. The OlyA6 assemblies closer to the membrane edge (Fig. 5*D*) appeared unstable during observation. The average observed height subsides toward the membrane edge caused by the large temporal fluctuations (Fig. 5*E*), resulting from the association and dissociation of OlyA6 from the stripes. The crystal growth was observed in both directions, longitudinal (extension of stripes) and transversal (orthogonal to stripe direction). Crystal extension in the longitudinal direction proceeded faster compared to the growth in the transversal direction.

**Figure 6:**
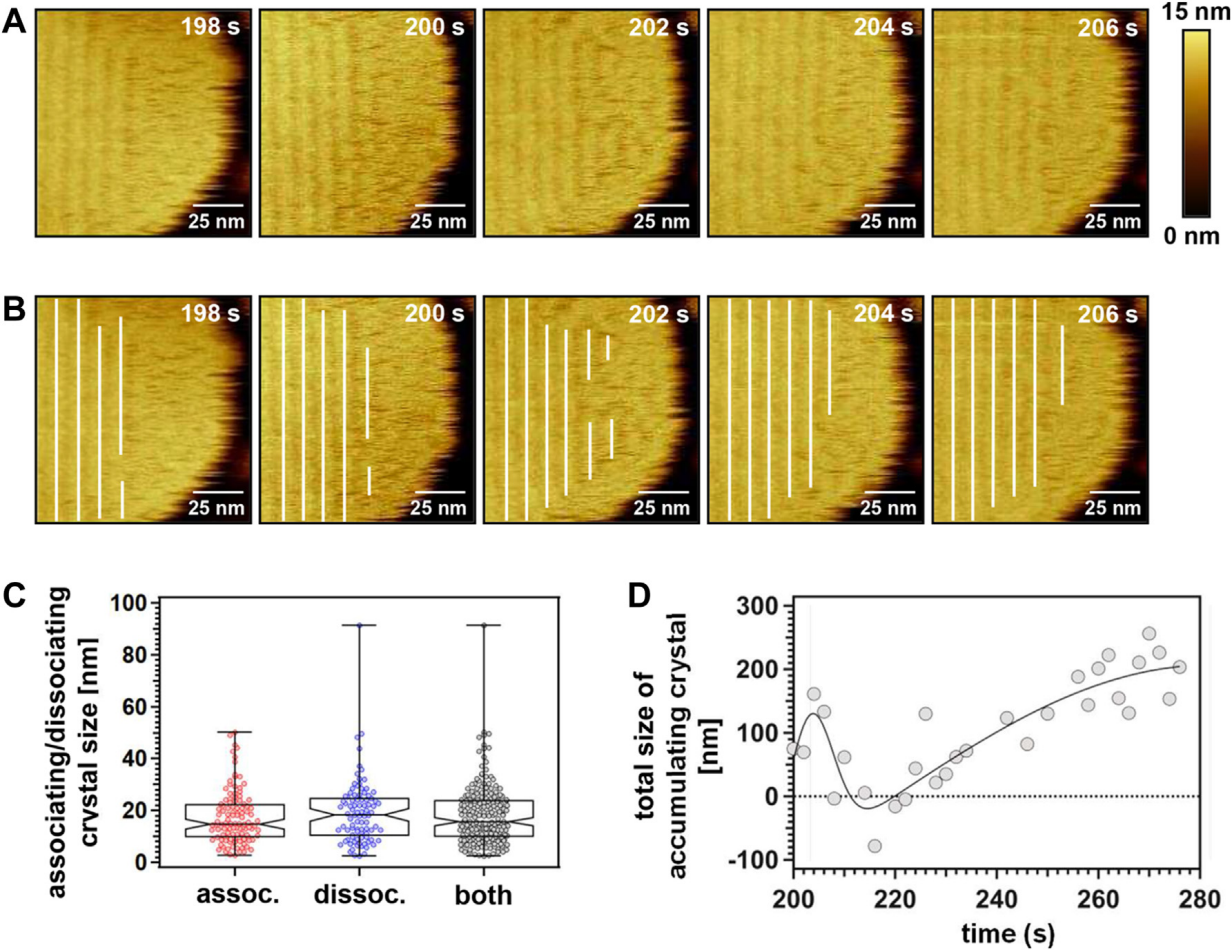
**Assembly dynamics of OlyA6 on regions close to CPE/Chol (1:1) membrane edge.***A*, HS-AFM height images showing the reorganization of OlyA6 close to the membrane edge. These HS-AFM images were recorded following those in Figure 4*A*. *B*, HS-AFM images in Figure 6*A* with *white lines* for OlyA6 crystal stripes. *C*, notched box plots showing the size distribution of the associating and dissociating OlyA6 units in the longitudinal direction with a 95% confidence interval of the median. The *center lines* indicate the median values, which are 14.7 (n = 113), 18.3 (n = 91), and 15.6 nm (n = 204) for association, dissociation, and both association and dissociation, respectively. Whiskers display the maximum and minimum size of the associating and dissociating units along each crystal stripe between the sequential frames. *D*, change in the total size of the accumulating crystal on the membrane from 200 s to 276 s. *Solid line*, Gaussian fit.

To gain a better understanding of the longitudinal crystal growth, the OlyA6 crystal stripes in higher resolution HS-AFM images were also marked with white lines (Fig. 6, *A* and *B*) and analyzed as demonstrated in Fig. S1. The statistical analysis (Fig. 6*C*) revealed that the associating and dissociating unit sizes populate at 14.7 nm and 18.3 nm, respectively. Considering that each image was captured every 2 s, the associating and dissociating unit size in shorter time intervals might actually be smaller than these values. Therefore, the smallest contributing unit size (∼2.5 nm) (Fig. 6*C*), which is similar to the lateral size of a monomer or a dimer rather than the significantly larger tetramer (23), may suggest that the longitudinal growth was facilitated by the incorporation of OlyA6 monomers or dimers rather than larger OlyA6 complexes. In addition, the analysis of the total size of the accumulating crystal stripes (Fig. 6*D* and Table S1) shows that the surface coverage increased over time. However, as the crystal stripes closer to the membrane edge were highly dynamic and continuously dissociated and re-associated, in some of the time intervals the total dissociation size was higher than the total association size, resulting in negative surface coverage and large fluctuations between 200 s to 222 s.

### OlyA6 crystal consists of hexameric repeat units as revealed by HS-AFM

**Figure 7:**
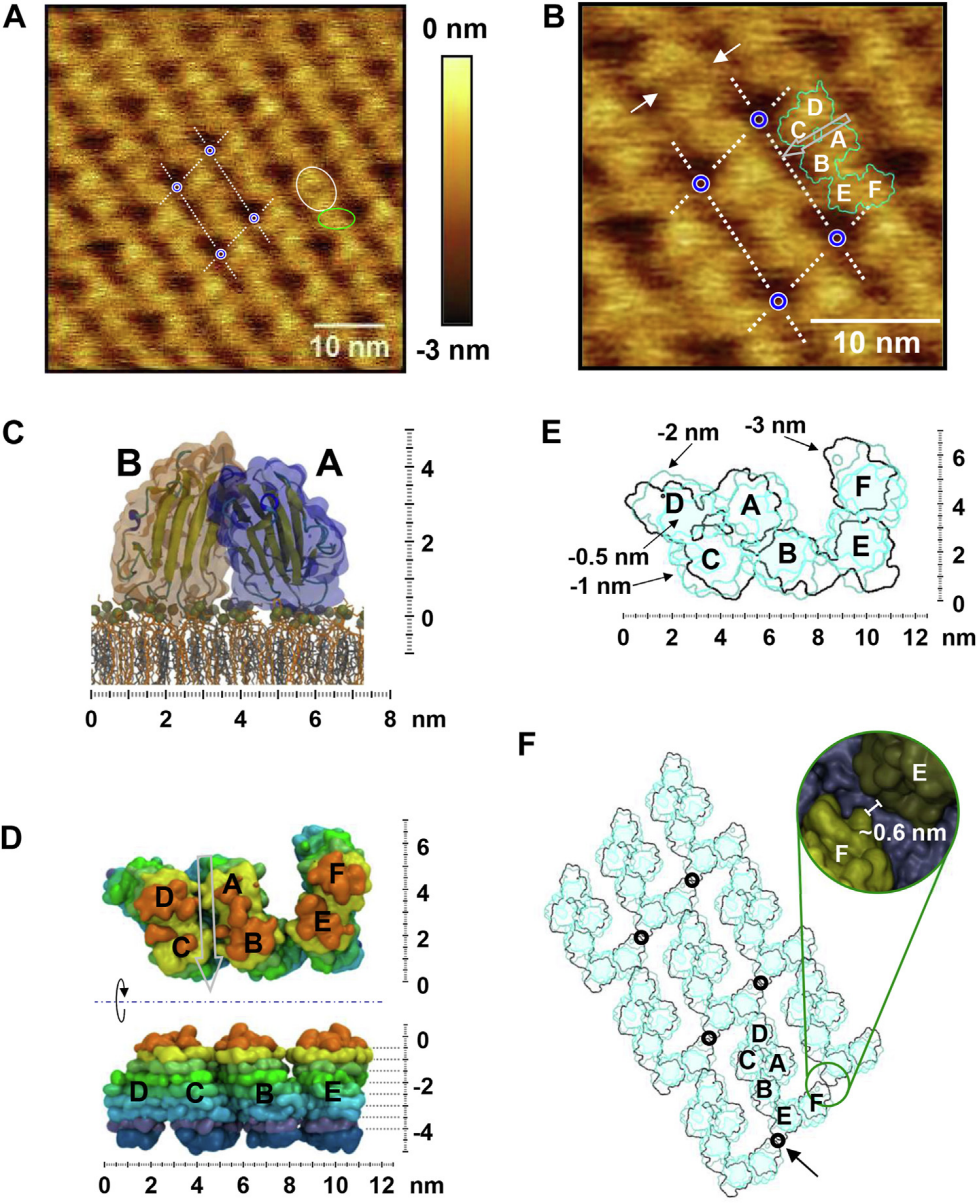
**OlyA6 crystalline arrangement.***A*, high-resolution AFM image of OlyA6 crystal assembly on the membrane. *White dashed box*, OlyA6 hexameric unit cell; *white circle*, head section composed of an OlyA6 tetramer; *green circle*, neck section comprised of an OlyA6 dimer. *B*, enlarged region of the image displayed in (*A*). *White dashed box* with *blue corners*, OlyA6 hexameric unit cell; *white arrows*, groove in OlyA6 tetramer; *grey arrow*, groove direction; *cyan outline*, OlyA6 hexameric repeat unit contour at −2.5 nm relative to protein top; *white letters*, naming of OlyA6 monomers. *C*, snapshot of MD simulation of OlyA6 dimer on the CPE/Chol membrane. *Golden sphere*, phosphate atom of CPE; *orange licorice*, carbon skeleton of CPE; *grey licorice*, carbon skeleton of Chol. *D*, *top* and *side* views of OlyA6 hexameric repeat unit on the membrane based on MD simulation. Each color corresponds to a 0.5 nm thick horizontal slice, while *top* and *bottom* slices are slightly thicker. Vertical height zero is set to the top of the protein backbone, excluding flexible C/N-terminal residues. *Gray arrow*, groove in the head section. *E*, *top* view with relief contours of OlyA6 hexameric repeat unit. Vertical height as indicated in (*D*, *side* view). *Light cyan area*, −0.5 nm contour; *cyan line*, −1 nm contour; *dark cyan line*, −2 nm contour; *black line*, −3 nm contour. *F*, contour map of reconstructed OlyA6 crystal arrangement. Contour colors as described in (*E*). Inset, enlarged closest approach between OlyA6 longitudinal crystal chains maintaining at least a single layer of water (*blue*) between monomers *F* and *E*. The minimum distance is ∼0.6 nm. *Black circle*, corner of unit cell; *black arrow*, closest approach between longitudinal OlyA6 chains.

Visualization of the stable assembly of OlyA6 on the d17:1/12:0-CPE/Chol bilayer by HS-AFM imaging revealed the defect-free structure of its 2D crystal at subnanometer resolution (Fig. 7*A*). The crystal stripes of OlyA6 resemble a pearl chain, comprising of repetitive three elliptical shapes of similar size. Two of the elliptical shapes appear to form an almost circular head section with a major axis of 6.5 ± 0.2 nm and a minor axis of 5.1 ± 0.2 nm (Fig. 7*A*, white circle). The third elliptical shape with a major axis of 5.1 ± 0.3 nm and a minor axis of 2.8 ± 0.2 nm (Fig. 7*A*, green circle), referred to as the neck section, attains a very different and seemingly slightly more variable orientation. The observed pattern is consistent with a primitive monoclinical lattice featuring an obtuse interaxial angle of γ = 100 ± 6 ° and unit cell vectors a = 10.8 ± 0.4 nm and b = 6.6 ± 0.3 nm (Fig. 7*A*, white dotted rectangle). The similar size of the unit cell vector b with the crystal stripe periodicity obtained from FFT analysis is consistent with the close packing of the OlyA6 crystal stripes.

**Figure 8:**
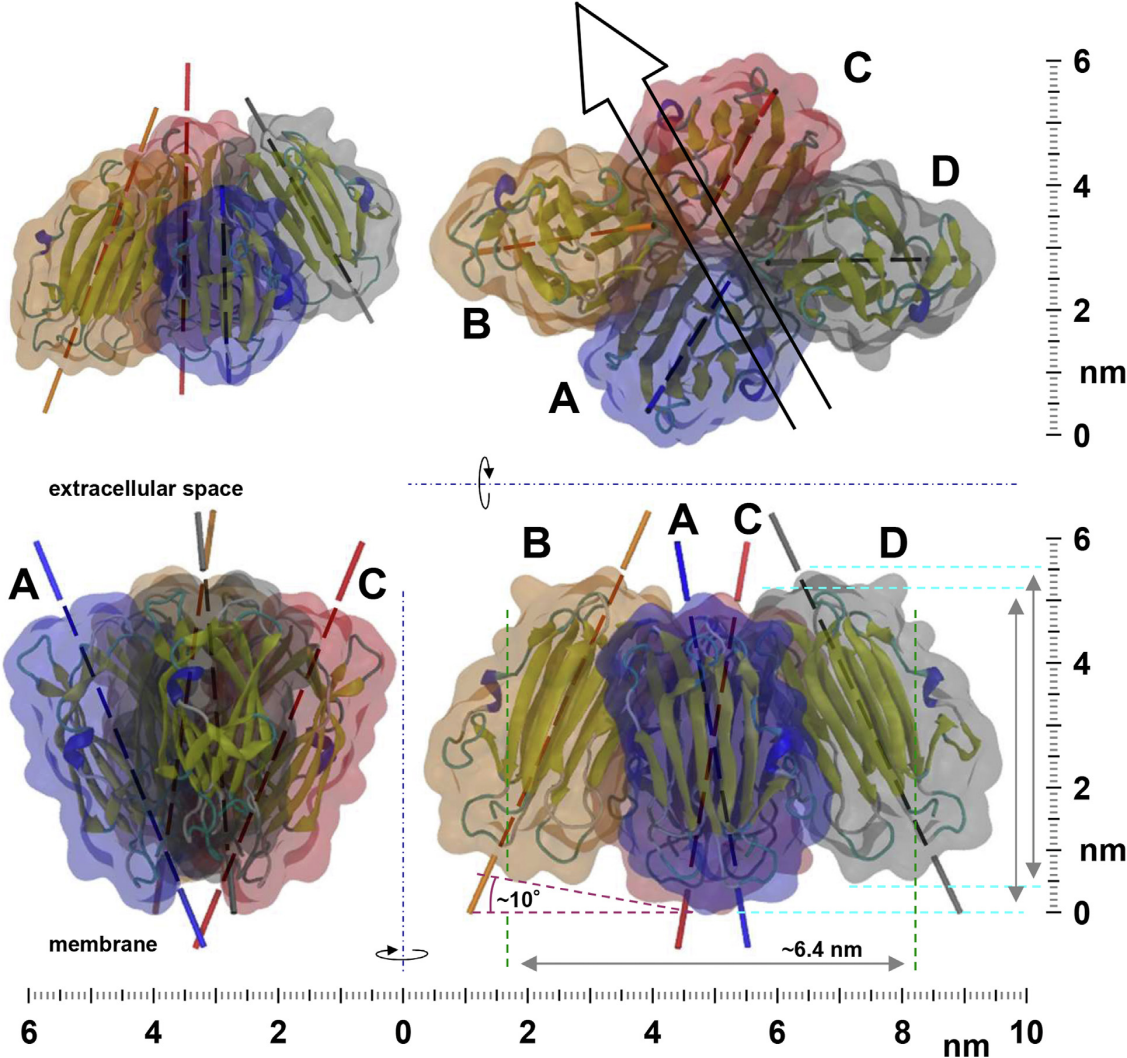
**Side and top views of OlyA6 tetramer (PDB ID: 6MYI).** Each subunit is represented by a space-filled model with a color matching the main axis. *Black arrow*, location of groove.

A closer look at the unit cell reveals that the head section exhibits a shallow groove, orthogonal to the chain direction (Fig. 7*B*, white arrows). The crystal structure of the OlyA6 tetramer, reported by Endapally *et al.* (15), exhibits a similar groove, virtually dividing the tetramer into two equal halves consisting of dimers AB and CD (Fig. 8, black arrow). The top view of the OlyA6 tetramer (Fig. 8) strongly resembles the head section of the linear OlyA6 arrangement (Fig. 7*B*, cyan outline). Due to the similar size of the neck section with each half of the head section, it seems reasonable to suggest that the neck section is comprised of an OlyA6 dimer, referred to herein as EF. As a result, high-resolution HS-AFM imaging revealed a unit cell composed of three OlyA6 dimers. The dimers assigned as AB, CD, and EF also correspond to the protrusions 2, 1, and 3 in Figure 3*E*. The height variation between these dimers is displayed at a higher lateral resolution in Fig. S2, revealing the elevation of EF in alternate hexameric repeat units, as mentioned earlier.

The formation of a hexameric repeat unit by three OlyA6 dimers suggests that a substantial fraction of the freely diffusing OlyA6 exists as dimers on the membrane surface rather than as tetramers. The similarity of unit vector a with the larger, more frequently observed longitudinal growth and dissociation steps (Fig. 6*C*) hints at higher stability of complete hexameric OlyA6 assemblies at the crystal edge compared to small OlyA6 assemblies. Nevertheless, the association and dissociation of complete hexameric OlyA6 assemblies within a time frame of 2 s is possible.

### MD reconstruction of OlyA6 hexameric arrangement reveals the structure of the membrane-bound OlyA6 dimer

The head and neck sections of the OlyA6 unit cell were derived from the tetrameric crystal structures of PlyA (23) and OlyA6 (15). The AB dimer in the head section readily tilted by ∼10° towards the membrane during initial MD simulation runs, fostering a stable membrane interaction for both subunits (Fig. 7*C*). Thus, this conformation was selected as the starting dimer conformation. The alignment of the tetramer, consisting of AB and CD dimers, was guided by the topology of the groove (Fig. 7*B*, white arrows). The groove across the head section is oriented at an angle of ∼90° relative to unit cell vector a and thus exhibits an offset of ∼10° compared to unit cell vector b. In this relative coordinate system, the membrane interface region is located at ∼-4.5 nm (Fig. 7*D*, side view). The contour of the OlyA6 hexameric repeat unit at the end of the production run above −2.5 nm (Fig. 7*D*, top view) is in good agreement with the experimental result (Fig. 7*B*, cyan outline). This suggests that the penetration of the AFM tip below −2.5 nm was somewhat obstructed by the packed environment. The contour maps of the hexameric repeat unit at different depths relative to the height of the top surface of the repeat unit are shown in Figure 7*E*. Reconstruction of the observed 2D crystal arrangement by periodic boundary conditions with the contour map of the OlyA6 hexameric repeat unit (Fig. 7*F*) is in good agreement with the high-resolution AFM data. This 2D crystal contour map suggests that below −2.5 nm the neck sections of adjacent pearl chains are very close to each other (Fig. 7*F*, black arrow). While the close proximity of the adjacent neck sections is likely responsible for the observed transversal chain distance, a single layer of water prevented direct protein–protein interaction between adjacent pearl chains (Fig. 7*F*, green circle and inset), throughout the production runs.

**Figure 9:**
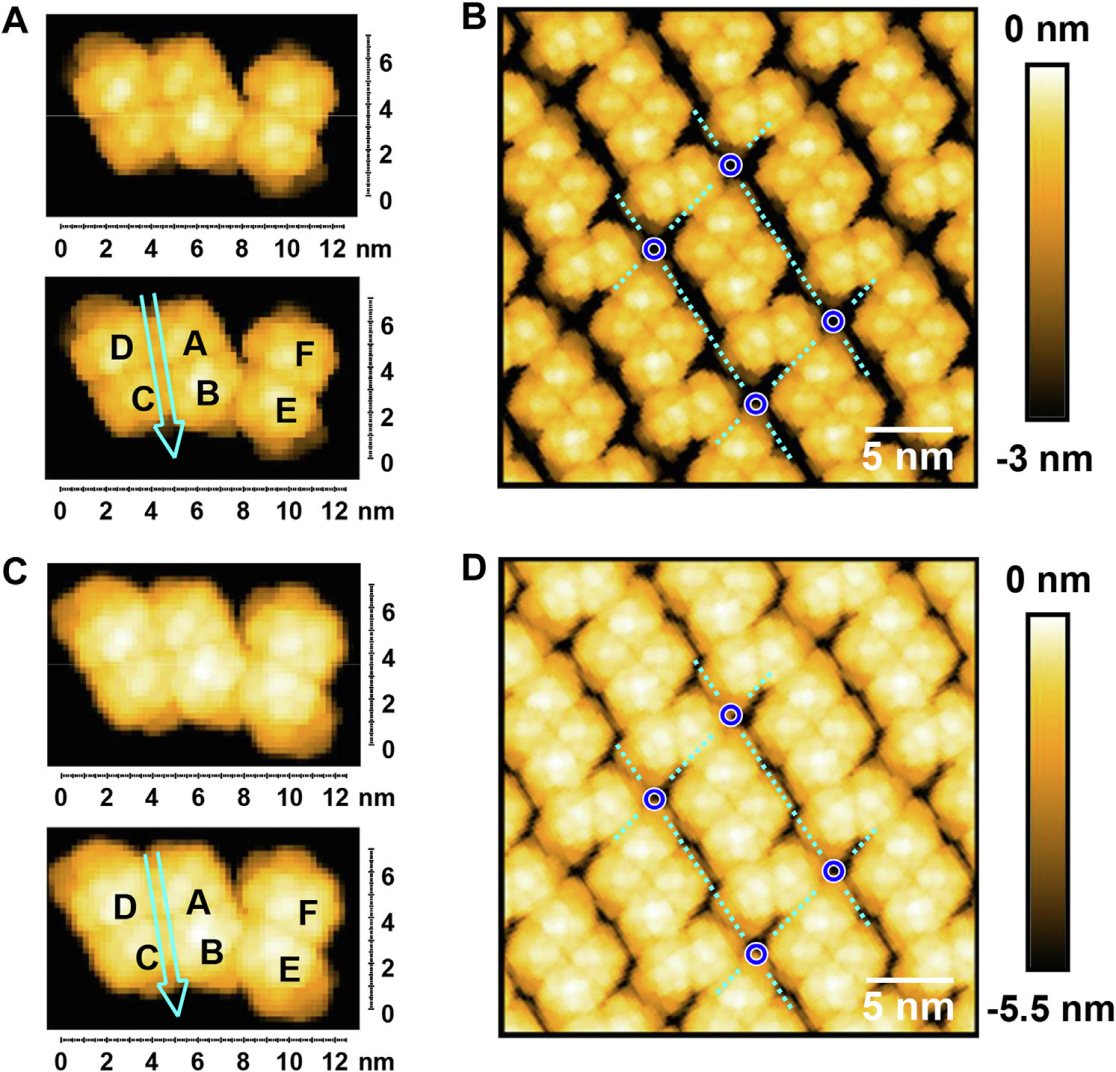
**Pseudo AFM image derived from final frame of OlyA6 hexameric repeat unit MD simulation.***A* and *C*, single OlyA6 repeat unit (*upper panel*) and with respective naming of OlyA6 monomers (*lower panel*). *Cyan arrow*, groove direction. Identical depth information for panels (*A* and *B*) as well as (*C* and *D*). *B and D*, 2D crystal arrangement of OlyA6 hexameric repeat unit. *Cyan dashed box* with *blue corners*, OlyA6 hexameric unit cell.

In addition to the MD reconstruction of the OlyA6 hexameric arrangement, pseudo AFM images were created to gain a better understanding of the topographical features of the OlyA6 crystal (Fig. 9). In Figure 7, *A* and *B*, there is an apparent height variation along the AB and CD dimers. Figure 9*A* clearly shows that one monomer in each dimer within the tetramer is lower than the neighboring monomer, that is, A is lower than B and C is lower than D, while the monomers in EF dimer exhibit a similar height, possibly resulting from their looser packing compared to those within the tetramer. MD simulation and pseudo AFM images also provide some insight into how closely the crystal stripes are packed. In Figure 7, *A* and *B*, the height range is from 0 to −3 nm due to the limited penetration of the AFM tip. The bottom contour of the OlyA6 crystal stripes is not visible in Figure 9*B*, which depicts the same height range as Figure 7, *A* and *B*. A more closely packed environment between adjacent crystal stripes appears below −3 nm (Fig. 9, *C* and *D*), with the closest contact formed by the EF dimers in the neighboring stripes.

The HS-AFM imaging showed that a gradual build-up of OlyA6 dimers on the membrane resulted in random but full coverage of the membrane with OlyA6, which gradually organized into a 2D crystal. High-resolution AFM imaging of the OlyA6 crystal showed a pearl chain arrangement with a primitive monoclinical lattice. Each unit cell is composed of six OlyA6 monomers. The neck section of the pearl chain is comprised of a single dimer, while the head section resembles the previously reported tetrameric crystal structures of PlyA (23) and OlyA6 (15). Tetramers, which are formed by OlyA6 dimers on the membrane, are likely to act as seeds for the 2D crystal arrangement. The closest distance between the neighboring pearl chains in the 2D crystalline OlyA6 arrangement was attained at the neck section of the neighboring chains. Thus, the transversal pearl chain distance was attributed to the packing of the neck section dimers in the neighboring chains. In contrast to the previously reported crystallization of lysenin, which showed the merging of individual crystalline domains into a defect-free crystal on the membrane surface (30), OlyA6 assembly took place by the continuous growth of one domain. The longitudinal crystal growth was likely dominated by the association/dissociation of OlyA6 dimers in the longitudinal direction, considering the smallest measured unit size of 2 nm within a frame time of 2 s.

## Conclusion

Our results demonstrate that membrane binding promotes the formation of the OlyA6 dimers, as OlyA6 exists as a monomer in bulk solution at the concentration used for HS-AFM imaging (Fig. S3). These dimers are functional units prone to either form the bi-component pores when combined with PlyB (3, 18) or cluster into larger OlyA6 assemblies as revealed herein. At high concentrations, OlyA6 arranges into defect-free, densely-packed 2D crystals. Considering that pore formation has been reported to be highly sensitive to the employed PlyA/PlyB or OlyA6/PlyB ratio (3, 18, 23), and specifically, an excess of OlyA6 was associated with suppressed hemolytic activity, it seems reasonable that membranes covered with tightly packed OlyA6 dimers do not have sufficient freedom to allow rearrangement to form pores with PlyB. Thus, the assembly state of OlyA6 on the membrane may regulate the cytotoxicity of the OlyA6/PlyB system.

## Experimental procedures

### Lipids

*N*-lauroyl-D-erythro-sphingosylphosphorylethanolamine (d17:1/12:0-CPE) was purchased from Avanti Polar Lipids Inc. (Alabama, AL). Cholesterol (Chol) was from Sigma (St Louis, MO).

### Proteins

An OlyA6 clone from a cDNA library prepared from the total mRNA of *P. ostreatus* (strain Plo5 from the ZIM collection of the Biotechnical Faculty, University of Ljubljana, Slovenia) (36) was used to construct the recombinant OlyA variants. The OlyA6 recombinant protein was produced as described previously (18). Protein size and purity were determined using SDS-PAGE (Bio-Rad) on homogeneous 12% acrylamide gels. The protein was then stained with SimplyBlue SafeStain (Thermo Fisher Scientific), or detected with anti-His antibodies after Western blotting (Qiagen).

### HS-AFM sample preparation and data acquisition

Multilamellar vesicles (MLV) of d17:1/12:0-CPE/Chol (1/1, mol/mol) were prepared in a solution of PBS (10 mM, pH 7.5) (Sigma) at a total lipid concentration of 1 mM. Unilamellar vesicles were prepared by sonication of MLVs at 20 kHz with an ultrasonic homogenizer, UH-50 from SMT, for 10 min. HS-AFM imaging was performed by NANOEXPLORER (Research Institute of Biomolecule Metrology Co., Ltd; RIBM) with cantilevers having carbon nanofiber (CNF; BL-AC10FS-A2) or electron beam deposited (EBD; BL-AC10EGS-A2) probes (Olympus Co). CNF probes have an effective length of 100 nm and the designed length for EBD probes was 600 nm. Both cantilevers have a spring constant of 0.1 N/m and a resonance frequency of around 500 kHz in water. The planar lipid bilayers, prepared by incubation of unilamellar vesicles on mica disk at 55 °C for 15 to 30 min, were inserted into PBS solution for HS-AFM imaging. After observation of the planar lipid bilayer in PBS solution at room temperature, 6.5 *μ*M OlyA6 was introduced into the imaging medium. The final concentration of OlyA6 in the imaging medium was around 0.35 *μ*M (5.8 *μ*g/ml).

### HS-AFM data analysis

Data processing was performed using Gwyddion (37) and Fiji (38). Gaussian filtering and 2D autocorrelation of the height images in Figure 3, *C* and *D* were performed by Gwyddion. The autocorrelation function was applied to enhance the periodic components as described by Stephant *et al.* (39). Contour maps were created using IGOR Pro (WaveMetrics, Lake Oswego, OR, USA). The longitudinal growth of the OlyA6 crystal stripes was calculated by measurement of the length of the white lines, drawn manually to trace the OlyA6 stripes. Fiji was used for length measurement. The lines tracing the OlyA6 crystal stripes in sequential images were overlapped to measure the increase (association) and decrease (dissociation) in the length of the same stripe between two sequential time frames. This procedure is demonstrated in Fig. S1 and was repeated for a total number of 31 sequential frames. The time interval between two sequential images was 2 s, however, some of the frames were omitted due to low image quality. After measuring the size of the associating and dissociating stripes between sequential frames, the total size of the accumulating crystal, *i.e.*, the surface coverage, was calculated by summing the crystal stripe size that accumulated at each frame relative to the preceding frame. The surface coverage values are listed in Table S1 and the calculation procedure is described in Table S1 legend. These values were used to plot Figure 6*D*. Statistical analysis and graphing were performed using IGOR Pro.

### MD OlyA6 model

Two suitable templates for OlyA6 model development were identified, namely PlyA (PBD id: 4OEB) and OlyA6 (PDB id: 6MYI). From a molecular dynamics point of view, both templates exhibit a highly similar conformation, as emphasized by an RMSD of ∼0.5 Å between the protein backbones of individual chains. The reported crystal structure of OlyA6 (15) features three point mutations, at residues S62C, A69E, and S95C, to facilitate crystal formation. The location of mutated residues is close to the putative binding and membrane interaction sites. In contrast, in the PlyA template, required point mutations were primarily facing away from the neighboring PlyA chain and located further away from the ligand or membrane binding region. Due to the less disturbed ligand binding region in PlyA compared to OlyA6, the model was based on the crystal structure of the PlyA tetramer (PDB id: 4OEB). All point mutations (K17Q, P26A, T39A, K45N, T49K, V50I, I51V, MSE113M) were introduced into each subunit of the tetramer with UCSF Chimera (version 1.11.2, build 41,376) and the orientation of the mutated residues mimic the orientation found in the OlyA6 crystal data. The missing residue at the N- (Met) and C-terminus (Gly-Asn) were created in PyMOL (version 1.8.6.1) and fused to each subunit of the tetramer using VMD (version 1.9.3). The protonation state of the tetramer was determined by H++ server (version 3.2, http://biophysics.cs.vt.edu/H++) at a pH of 6.5, suggesting a total charge of +1 for each subunit. The subunit numbering is as indicated in Figure 7*B*.

### MD CPE model

The palmitoyl ceramide phosphoethanolamine (residue: PCPE) was derived from palmitoyl sphingomyelin (residue: PSM) by replacing the choline head group with ethanolamine from dipalmitoyl phosphatidylethanolamine (residue: DPPE). Both residues are part of the CHARMM36 lipid and sphingomyelin force field. The atom type of the ethylene group in both head groups is identical, thus no new parameters were required.

### MD membrane setup

The pure PCPE bilayer, composed of 36 PCPE residues per leaflet, was equilibrated for 200 ns, yielding 46.5 ± 0.4 Å^2^ as the average area per lipid and a thickness of 4.37 ± 0.04 nm. The equilibrated PCPE:Chol = 1:1 bilayer, composed of 36 PCPE and 36 Chol residues per leaflet, yielded an average area per lipid of 39.4 ± 0.2 Å^2^ and a thickness of 4.54 ± 0.03 nm, consistent with a limited condensing effect of ∼3 Å^2^. The resulting membrane patch was used as starting point for all OlyA6–lipid interaction simulations.

### MD OlyA6 dimer and tetramer

OlyA6 dimer containing the desired monomers was extracted from the parametrized OlyA6 tetramer model. In general, OlyA6 dimer or tetramer placement above the equilibrated PCPE:Chol = 1:1 patch was adjusted to a distance of ∼0.5 nm between the amide nitrogen of the conserved Pro95 and the average height of the lipid phosphate groups of the bilayer. The placement of Pro95 close to the membrane interface was based on its reported proximity to the lipid binding site (10, 15). The position with the least conflicts between protein residues and lipid head groups was selected by lateral adjustments and any remaining conflicts were manually resolved by rotating the lipid head groups away from the conflict zones. Harmonic distance restraints between protein and membrane residues were applied during the initial 10 ps to ensure successful attachment. The membrane patch was enlarged by multiplying the equilibrated membrane section and lateral displacement as needed. After 80 ns equilibration was followed by a 20 ns production run.

### MD OlyA6 hexameric repeat unit

OlyA6 hexameric repeat unit was generated by duplicating chains A and B of the tetramer, renamed to chains E and F and placed according to HS-AFM results. Conflicts between neighboring dimers were minimized primarily by adjusting the shift and rotation of the duplicated dimers. The membrane patch was created by copying the equilibrated PCPE:Chol = 1:1 patch in X and Y directions. A total of 180 lipids per leaflet were determined as the best fit for the experimental unit cell size. The unit cell vector a was oriented along the X-axis. Each dimer subunit of the hexameric unit cell was placed to satisfy a distance of ∼0.5 nm between the membrane section and the conserved Pro95. Any spatial conflicts were resolved manually by rotation of the affected lipid head groups. Harmonic distance restraints between selected protein and membrane residues were applied during the initial 5 ns of the equilibration run. A total of 50 ns equilibration was followed by a 50 ns production run. To reduce bias due to the selected starting position, each of the 5 runs featured a slightly different initial position of the OlyA6 hexameric repeat unit relative to the membrane patch.

### MD general conditions and software

All models were parameterized with the CHARM36 force field for proteins, lipids, and general force field (CGenFF). Initial assembly and post-processing were performed with VMD (version 1.9.3), utilizing proprietary scripts. Molecular dynamics simulations were executed in the NPT ensemble with NAMD (version 2.10 with CUDA acceleration) utilizing 2 fs steps, 1 atm pressure, a temperature of 312 K, and flexible simulation cells with periodic boundary conditions. All simulations were fully hydrated with a significant excess of water and neutralized with physiological potassium chloride concentration. Unless stated otherwise, no restraints were applied during equilibration and production runs. Bulk parameters were averaged over the whole production run unless stated otherwise.

### Pseudo AFM image generation

The OlyA6 PDB structure information was extracted from the last frame of MD simulation 5 in VMD (version 1.9.3). Single OlyA6 hexameric repeat unit pseudo AFM images were created with BioAFMviewer (version 2.5) (40, 41) at a scan step of 0.25 nm, cone angle of 8° and tip radius of 0.5 nm. Pseudo AFM images of the 2D crystalline arrangement were created by copying the PDB structure information of the simulated OlyA6 repeat unit into a 4 by 7 array according to the periodic boundary dimensions of the associated MD simulation frame using VMD. The resulting PDB structure information of the 2D crystalline arrangement was visualized with BioAFMviewer (version 2.5) at a scan step of 0.25 nm, cone angle of 8° and tip radius of 0.5 nm.

### Sedimentation velocity analytical ultracentrifugation

Sedimentation velocity experiments were conducted in a ProteomeLab XL-I analytical ultracentrifuge (Beckman Coulter) at 20 °C. The OlyA6 samples in AUC buffer (10 mM Tris pH 7.5, 20 mM NaCl) were loaded into AUC cell assemblies with 12 or 3 mm charcoal-filled Epon double-sector centerpieces. The sample cells were loaded into a four-hole An-60 Ti rotor for temperature equilibration for 2 to 3 h, followed by an acceleration to full speed at 50,000 RPM. Absorbance data at 280 nm (for concentrations at 0.3 and 1.2 mg/ml) and 231 nm (for concentration at 0.1 mg/ml) were collected at 3 min intervals for 15 h. The partial specific volume of the protein, buffer density, and viscosity were calculated using the software SEDNTERP. Sedimentation data were time corrected and modeled with diffusion-deconvoluted sedimentation coefficient distributions *c(s)* in SEDFIT 16.1c, with signal-average frictional ratio and meniscus position refined with nonlinear regression (42). Maximum entropy regularization was applied at a confidence level of 68%. Sedimentation coefficient distributions were corrected to standard conditions of 20 °C in water (*s*_*20,W*_). The plot was created in GUSSI (43).

## Data and materials availability

The datasets generated and/or analyzed during the current study are available from the corresponding author on reasonable request.

## Supporting information

This article contains supporting information.

## Conflict of interest

The authors declare that they have no conflicts of interest with the contents of this article.
