# Supplementary material for: Assembly dynamics and structure of an aegerolysin, ostreolysin A6
Source: J Biol Chem. 2023 Jun 19;299(8):104940. doi: 10.1016/j.jbc.2023.104940 (PMC10366546; doi:10.1016/j.jbc.2023.104940)
Supplement: Supporting information [file mmc1.pdf]

Supporting Information

Assembly dynamics and structure of an aegerolysin, ostreolysin A6

Neval Yilmaz[1,2]*, Anastasija Panevska[3], Nario Tomishige[1,4], Ludovic Richert[4], Yves Mély[4], Kristina Sepčić[3], Peter Greimel[1]* and Toshihide Kobayashi[1,4]*

[1]Lipid Biology Laboratory, RIKEN, 2-1, Hirosawa, Wako, Saitama 351-0198, Japan
[2] NanoLSI, Kanazawa University, Kakuma-machi, Kanazawa, Ishikawa 920-1192, Japan
[3]Department of Biology, Biotechnical Faculty, University of Ljubljana, Jamnikarjeva 101, 1000, Ljubljana, Slovenia
[4]Laboratoire de Bioimagerie et Pathologies, UMR 7021 CNRS, Université de Strasbourg, Faculté de Pharmacie, 74 route du Rhin, 67401 Illkirch, France

***Corresponding Authors:**

Neval Yilmaz (nevaly@staff.kanazawa-u.ac.jp)
Peter Greimel (petergreimel@riken.jp)
Toshihide Kobayashi (toshihide.kobayashi@unistra.fr)

Table S1. Total size of associating and dissociating units between each frame and the increase in surface coverage with time.

f	Time (s)	Association (a_{total}) (nm)	Dissociation (d_{total}) (nm)	Surface coverage (nm)
1	198	-	-	-
2	200	75	0	75
3	202	49	54	70
4	204	111	19	161
5	206	0	28	133
6	208	5	142	-4
7	210	65	0	62
8	214	42	98	5
9	216	13	97	-78
10	220	81	18	-16
11	222	86	75	-5
12	224	135	86	44
13	226	186	101	130
14	228	67	175	22
15	230	88	75	35
16	232	95	68	62
17	234	47	38	72
18	242	69	17	123
19	246	37	78	82
20	250	71	24	130
21	256	109	50	188
22	258	0	45	144
23	260	89	32	201
24	262	54	32	222
25	264	34	102	154
26	266	41	64	131
27	268	95	16	211
28	270	60	14	256
29	272	31	61	226
30	274	41	114	153
31	276	83	33	203

Surface coverage is the total size of the accumulating unit on membrane starting from the reference time point (f=1). Surface coverage at frame "f" is defined as $(a_{total}-d_{total})_{f-1}+(a_{total}-d_{total})_f$, where $a_{total} = a_1 + a_2 +\ldots+ a_i$ and $d_{total} = d_1 + d_2 +\ldots+ d_i$ as demonstrated in **Fig S1**. Some of the frames between 198 s and 276 s were not included because of the low image quality.

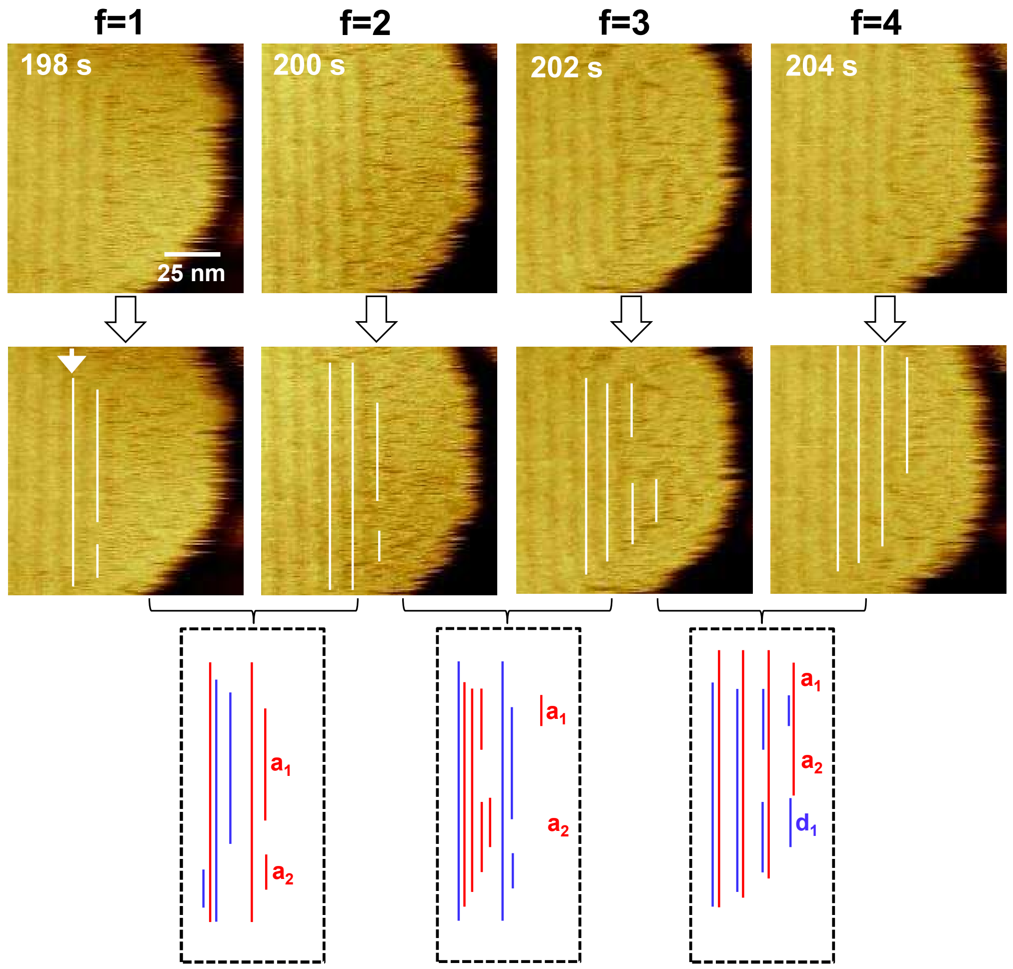

Associating and dissociating unit size between each sequential frame (f=1….31)

Association (nm)	Dissociation (nm)
a_1	d_1
a_2	d_2
.	.
.	.
.	.
a_i	d_i

Figure S1. Procedure to calculate the associating and dissociating unit size. The HS-AFM images in **Fig 6a** were re-used to demonstrate the calculation procedure. The white lines on the HS-AFM images in the second row trace the OlyA6 crystal stripes. The lines at 198 s were overlapped with those at 200 s. The lines at 198 s and 200 s in the overlay are shown in blue and red, respectively, in the dashed line box. The same procedure was repeated for the remaining 30 frames. Here, the first four frames (f=1-4) are shown to demonstrate the procedure to measure the associating and dissociating unit size between each sequential frame. The associating and dissociating units are indicated as "a_i" and "d_i", respectively, where a and d are the unit length and i is the number of units measured separately for association and dissociation between sequential frames. Although the changes in line length between two sequential images were measured starting from the white line indicated by the white arrow, here only the lines nearest to the membrane edge are marked "a_1, a_2, and d_1" for demonstration.

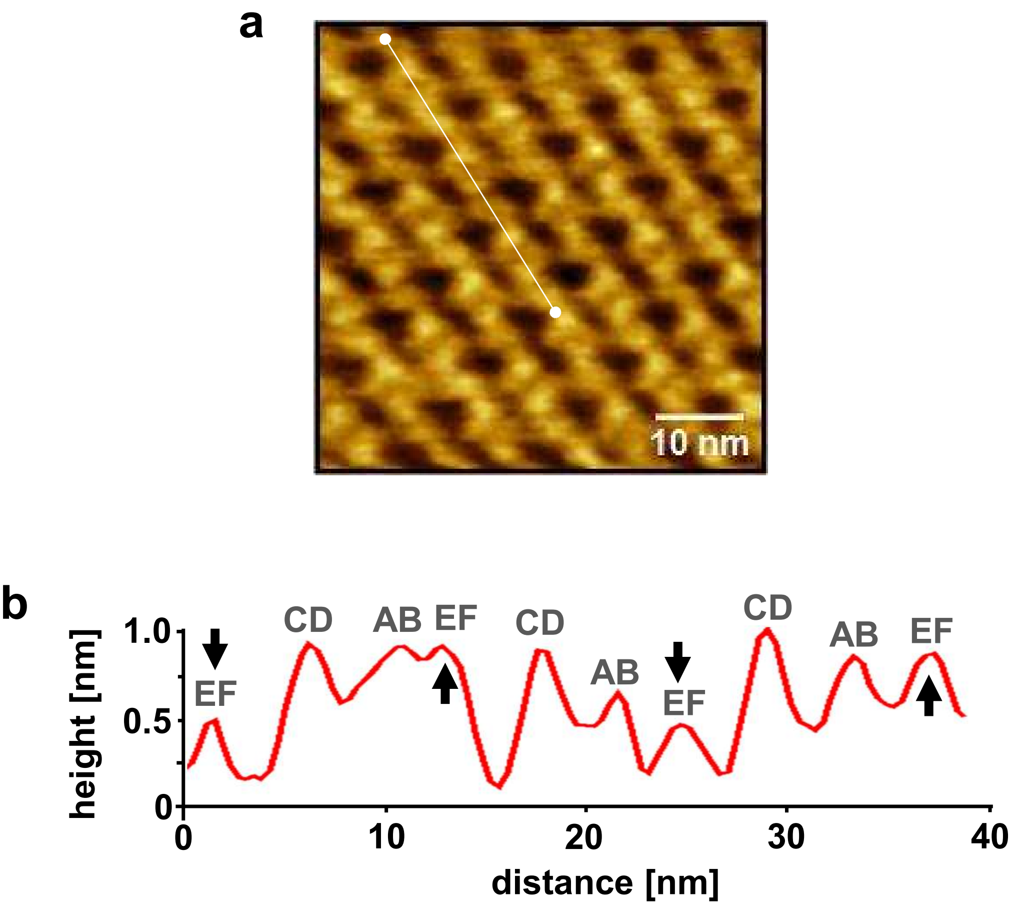

Figure S2. Change in height along the OlyA6 crystal stripe. (**a**) The HS-AFM height image shown in **Fig 7a** after Gaussian filtering. Gaussian filtering was applied to reveal the protrusions of AB, CD and EF dimers more clearly. (**b**) Height profile along the white line in **a**.

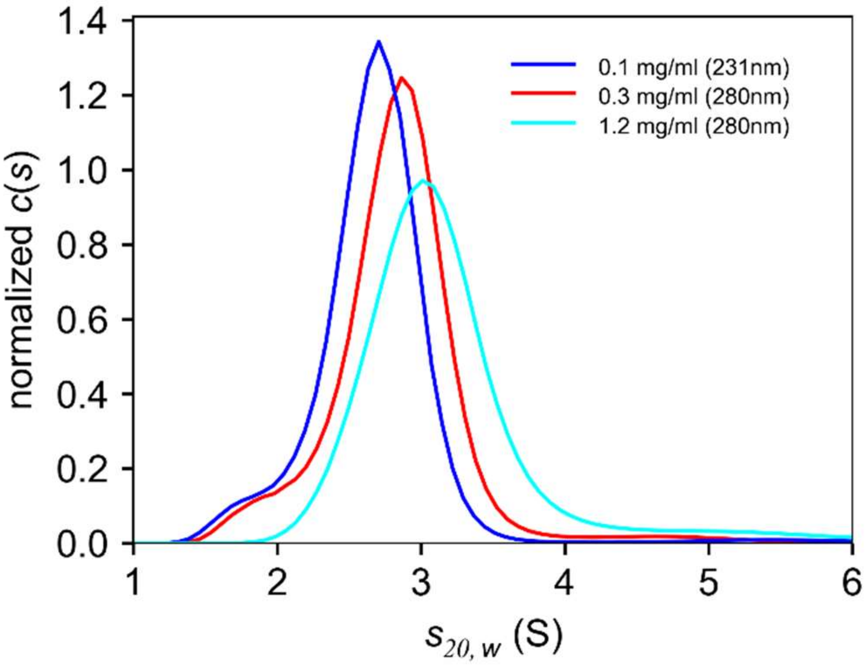

Figure S3. Sedimentation coefficient distributions *c(s)* determined from analysis of absorbance sedimentation velocity data for concentrations of OlyA6 over the range of 0.1 to 1.2 mg/ml. A major large peak can be discerned which shifts to higher sedimentation coefficients as the concentration increases. Calculated *s* values for spherical proteins of 17.5 and 35 kDa, with no bound water, are 2.5 and 3.9 S, respectively. These are the maximum possible *s* values for the OlyA6 monomer and dimer. The observation of broad, concentration-dependent peaks at ~2.6-3 S is thus consistent with a rapidly reversible monomer-dimer association of OlyA6.

Movie Legend

Movie 1. HS-AFM movie showing the crystallization of OlyA6 on CPE-containing planar membrane. Original scan size was 200 nm × 200 nm. Frame size was reduced to 191 nm × 170 nm after drift correction and cropping. Scan speed was 2 seconds per frame.
